# Human Biomonitoring Data Enables Evidence-Informed Policy to Reduce Internal Exposure to Persistent Organic Compounds: A Case Study

**DOI:** 10.3390/ijerph18115559

**Published:** 2021-05-22

**Authors:** Ann Colles, Dries Coertjens, Bert Morrens, Elly Den Hond, Melissa Paulussen, Liesbeth Bruckers, Eva Govarts, Adrian Covaci, Gudrun Koppen, Kim Croes, Vera Nelen, Nicolas Van Larebeke, Stefaan De Henauw, Tine Fierens, Griet Van Gestel, Hana Chovanova, Maja Mampaey, Karen Van Campenhout, Ilse Loots, Willy Baeyens, Greet Schoeters

**Affiliations:** 1 VITO Health, Boeretang 200, 2400 Mol, Belgium; eva.govarts@vito.be (E.G.); gudrun.koppen@vito.be (G.K.); greet.schoeters@vito.be (G.S.); 2 Department of Sociology, Faculty of Social Sciences, University of Antwerp, Sint-Jacobstraat 2, 2000 Antwerp, Belgium; dries.coertjens@uantwerpen.be (D.C.); bert.morrens@uantwerpen.be (B.M.); ilse.loots@uantwerpen.be (I.L.); 3 Provincial Institute of Hygiene, Kronenburgstraat 45, 2000 Antwerp, Belgium; Elly.DENHOND@provincieantwerpen.be (E.D.H.); melissa.paulussen@hvrt.be (M.P.); vera.NELEN@provincieantwerpen.be (V.N.); 4 i-BioStat, Data Science Institute, Hasselt University, Martelarenlaan 42, 3500 Hasselt, Belgium; Liesbeth.bruckers@uhasselt.be; 5 Toxicological Centre, University of Antwerp, Universiteitsplein 1, 2610 Antwerp, Belgium; adrian.covaci@uantwerpen.be; 6 Analytical, Environmental and Geo-Chemistry (AMGC), Vrije Universiteit Brussel, Pleinlaan 2, 1050 Brussels, Belgium; kim.croes@ugent.be (K.C.); nicolas.vanlarebeke@ugent.be (N.V.L.); wbaeyens@vub.be (W.B.); 7 Department of Public Health and Primary Care, Ghent University, C. Heymanslaan 10, 9000 Ghent, Belgium; stefaan.dehenauw@ugent.be; 8 Flanders Environment Agency (VMM), Dr. De Moorstraat 24, 9300 Aalst, Belgium; t.fierens@vmm.be; 9 Public Waste Agency of Flanders (OVAM), Stationsstraat 110, 2800 Mechelen, Belgium; griet.van.gestel@ovam.be; 10 Flemish Ministry of Welfare, Public Health and Family, Flemish Agency for Care and Health, Environmental Health Section, Koning Albert-II laan 35 bus 33, 1030 Brussels, Belgium; hana.chovanova@vlaanderen.be; 11 Department of Environment & Spatial Development, Flemish Planning Bureau for the Environment and Spatial Development, Koning Albert-II laan 20 bus 8, 1000 Brussels, Belgium; maja.mampaey@vlaanderen.be (M.M.); karen.vancampenhout@vlaanderen.be (K.V.C.)

**Keywords:** POPs, PCBs, dioxins, human biomonitoring, science-to-policy, participation

## Abstract

Human biomonitoring (HBM) monitors levels of environmental pollutants in human samples, which often is a topic of concern for residents near industrially contaminated sites (ICSs). Around an ICS area in Menen (Belgium), including a (former) municipal waste incinerator and a metal recovery plant, increasing environmental concentrations of dioxins and polychlorinated biphenyls (PCBs) were observed, causing growing concern among residents and authorities. The local community succeeded in convincing the responsible authorities to investigate the problem and offer research funding. Persistent organic pollutants (POPs) were measured in two consecutive HBM studies (2002–2006 and 2010–2011), in the context of the Flemish Environment and Health Study (FLEHS), as well as in soil and locally produced food. Meanwhile, local authorities discouraged consumption of locally produced food in a delineated area of higher exposure risk. Ultimately, HBM and environmental data enabled tailored dietary recommendations. This article demonstrates the usefulness of HBM in documenting the body burdens of residents near the ICS, identifying exposure routes, evaluating remediating actions and providing information for tailored policy strategies aiding to further exposure reduction. It also highlights the role of the local stakeholders as an example of community-based participatory research and how such an approach can create societal support for research and policy.

## 1. Introduction

Human biomonitoring (HBM) measures environmental chemicals, or their breakdown products (metabolites), in human samples, such as blood and urine. These exposure biomarkers can be complemented by biomarkers that indicate early biological responses and health outcomes. Such a molecular epidemiological approach allows the detection of risks to public health with a far greater sensitivity than classical epidemiology. HBM aggregates exposure to environmental chemicals from different sources and exposure pathways [1]. When applied to residents in industrially contaminated sites (ICSs), HBM provides complementary information to the often scattered environmental data (e.g., in soil, crops, atmospheric deposition). HBM answers the question to what extent environmental pollution has entered the human body [2], which is often a real concern of residents near an ICS. In this article, we describe a case study of an ICS in which HBM was successfully used as a basis for evidence-informed policy making and to address the concerns of the local community.

In the area of Menen, in Flanders (Belgium), an ICS is situated on the Belgian/French border, adjacent to residential areas. On the Belgian side of the border, the ICS housed a municipal waste incinerator (MWI) until 2005, as well as a large metal recycling plant, which is still active. Both industrial activities, municipal waste incinerating and metal recycling, are also present on the French side of the border. Waste incinerators and metal recycling industries (e.g., electronic waste and car shredder waste) are generally known as major sources of environmental contamination with polychlorinated dibenzo-p-dioxins (PCDDs), dibenzofurans (PCDFs) and dioxin-like polychlorinated biphenyls (DL-PCBs) [3]. During incineration of municipal solid waste, PCDD/Fs and polychlorinated biphenyls (PCBs) can be formed in the combustion process when chlorine containing materials are present [4,5]. The Waste Incineration Directive, published in 2000, restricted emissions of PCDD/Fs to air to a value of 0.1 ng Toxicity Equivalents (TEQ)/m^3^ [6]. In metal recycling plants, the waste fractions can be very diverse. Thermal processes used for separating different scrap fractions can be sources of PCDD/Fs and PCBs [7,8]. PCDD/Fs and PCBs are persistent organic pollutants (POPs) with endocrine disruptive properties and well-known adverse effects in humans on reproductivity, the immune system and neurodevelopment [9,10]. The International Agency for Research on Cancer (IARC) classified congener 2,3,7,8-tetrachloro-p-dibenzodioxin and PCBs as “known human carcinogen” [11,12].

The Flanders Environment Agency monitors ambient air quality near MWIs. Deposition of PCDD/Fs has been measured since 1995, supplemented with PCB 126 depositions since 2002. Near the MWI in Menen, exceedances of Flemish deposition guidelines for PCDD/Fs [13] have repeatedly been reported [14]. Even after closing the MWI in 2005, high deposition levels of PCDD/Fs (over 100 pg TEQ/m^2^.day) and PCB 126 (over 250 pg TEQ/m^2^.day) were frequently observed. These observations were in contrast to the decreasing trends in other Flemish monitoring stations. Measurements of PCDD/Fs and PCBs in soil, vegetables and home-produced eggs from the residential areas near the ICS of Menen showed elevated levels compared to background locations [15]. Using these data in dietary exposure calculations with the Xtrafood model identified consumption of home-produced chicken eggs to be a major contributor to the PCDD/F exposure of residents (dietary exposure of 26 pg I-TEQ/day via eggs with possible increase to 84 pg I-TEQ/day). Food of animal origin is generally considered to be a dominant contributor to PCDD/Fs and PCBs exposure in humans [16]. In this context, the Flemish and local authorities delineated an area within the city of Menen in 2003, in which consumption of locally produced food was discouraged, further referred to as the precautionary area. Citizens with residence within the precautionary area were advised not to consume milk from local farms or home-produced chicken eggs, and to remove the outside leaves and thoroughly wash locally produced vegetables before consumption.

Meanwhile, the Flemish government issued a Decree on Preventive Health Care in 2003, in which environmental health was recognized as an important field for preventive health action. It also formed a legal basis for a Flemish human biomonitoring program, i.e., the Flemish Environment and Health Study (FLEHS), as a scientific basis for evidence-informed policy. High concerns regarding human exposure and public health in the PCDD/Fs and PCBs contaminated area near the MWI of Menen resulted in two subsequent HBM studies in the region of Menen as part of the FLEHS program and an additional study on POP levels in gardens and home-produced eggs. In this context, we describe how human biomonitoring has been used as a policy instrument to investigate to what extent environmental pollution is contaminating the human body of residents, to follow up exposure over time, to explore effectiveness of policy measures and awareness raising actions and to develop policy actions to meet the needs of local residents. We also discuss the role of the local community and stakeholders as an example of community-based participatory research and how it stimulated the dialogue between the community and policy makers. FLEHS studies in Flanders are explicitly embedded in a multi-disciplinary research consortium in which social, communicative and governance aspects are part of the research design.

## 2. Materials and Methods

**Figure 1:**
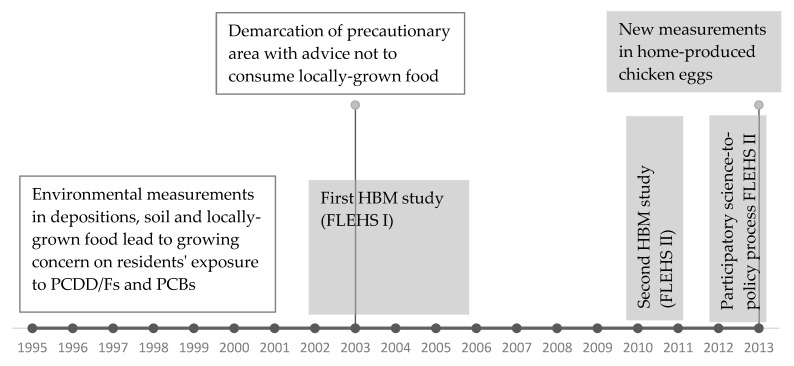
Time-flow of the sequence of research activities on polychlorinated dibenzo-p-dioxins and furans (PCDD/Fs) and polychlorinated biphenyls (PCBs) in the region of Menen. Activities in the grey boxes are addressed in this article. FLEHS = Flemish Environment and Health Study.

This section describes the methodology used in both FLEHS HBM studies in the region of Menen and in the study on POPs in home-produced chicken eggs. In this article, we reconstruct the sequence of study results on POPs in the region of Menen that shaped decision making and public perception and finally resulted in tailored dietary recommendations on safe consumption of home-produced chicken eggs. The time-flow of these research activities is presented in Figure 1, with the activities addressed in this article in the grey boxes. The concept of the FLEHS studies and overall results have been published in separate publications. This article focusses specifically on the Menen area.

Data from environmental samples and human biomonitoring were provided with a unique identification code in order to pseudonymize results and data.

### 2.1. Study Area

**Figure 2:**
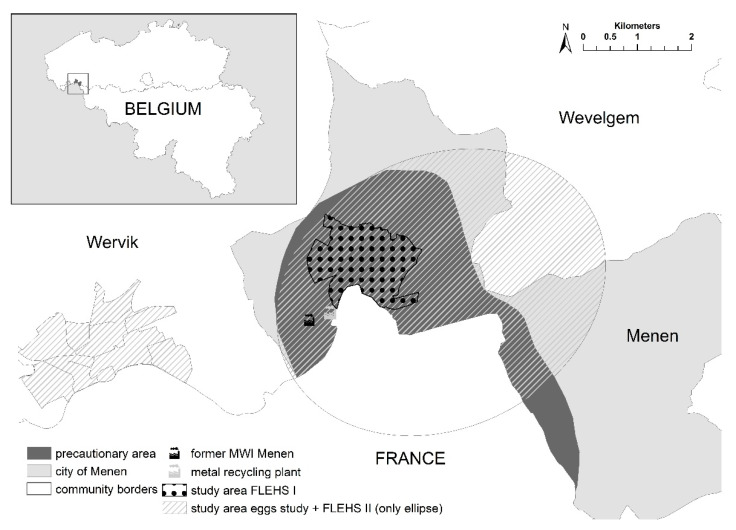
Geographic location of the FLEHS I study area (dotted area), the FLEHS II study area (Flemish territory within the ellipse), the study area of the eggs study (Flemish shaded areas in the ellipse and in Wervik), the precautionary area (dark grey), the (former) municipal waste incinerator (MWI) and the metal recycling plant in the region of Menen, Flanders, Belgium.

The study area of both HBM studies near the MWI of Menen was delineated based on modelled geographic emission distribution using a bi-Gaussian plume model (Immission Frequency Distribution Model, IFDM) [17]. In the FLEHS I study (sampling period between October 2002–February 2004 (newborns), October 2002–July 2004 (adolescents) and September 2004–June 2005 (adults)), the study area was restricted to residential areas close to the industrial activities (dotted area in Figure 2). The study area was expanded in the FLEHS II study (sampling period May 2010–February 2011) to include the Flemish territory inside an elliptical area (ellipse in Figure 2, longest diameter is 6.3 km) with the ICS as one of the focal points and orientation according to the dominant wind direction (excluding French territory for administrative reasons). The expansion was based on environmental data (emission inventory, plume calculations, deposition data, measurements in soil, groundwater, sediments, eggs, vegetables and milk) on the presence of additional and smaller industrial sources outside the ICS and on information received from local stakeholders. Home-produced chicken eggs were collected in an expanded study area to meet the requests of local stakeholders. In addition to the elliptical area, a residential area in the neighboring community (Wervik, Figure 2) was included, where another small metal recycling company was situated. The precautionary area in which the local authorities have discouraged consumption of home-produced food since 2003 is also shown in Figure 2 (dark grey).

### 2.2. HBM Studies

Table 1 presents an overview of the different study populations and number of participants in the FLEHS I and II studies, for the reference groups as well as for the region of Menen. The first cross-sectional HBM study in Menen was organized as part of the first FLEHS cycle (2002–2006). In FLEHS I, nine biomarkers of exposure and 40 biomarkers of effects were assessed in three age groups of the general population (newborns, 14–15 year-old adolescents and 50–65 year-old adults), recruited in eight types of areas in Flanders with different environmental profiles [18]. One of these eight types of area consisted of residential areas near municipal waste incinerators, among which was the study area near the MWI in Menen. In Menen 14 newborns, 14 adolescents and 35 adults were enrolled, of whom (cord) blood and urine samples were collected by trained study nurses.

The second cross-sectional HBM study in the region of Menen was organized as part of the second FLEHS cycle (2007–2011). In FLEHS II, Flemish reference values were established for a large set of biomarkers (40 biomarkers of exposure and 32 biomarkers of effects), measured in three age groups of the general population (newborns, 14–15 year-old adolescents and 20–40 year-old adults) recruited as a representative study population for Flanders using a stratified multi-stage design [19]. Additionally, a HBM case study was organized near the ICS of Menen between May 2010 and February 2011, where 199 adolescents (14–15 years old) were enrolled at secondary schools, supplemented with home-visits supported by local community members. Samples of morning urine, hair and serum were collected by trained study nurses. Choosing a study population in FLEHS II within the same age range as included in FLEHS I facilitated comparison of study results. Selecting adolescents as a target population near an ICS has the advantage that occupational exposure is negligible.

In both HBM studies, inclusion criteria were (i) residing in the study area for at least 5 years prior to the study, (ii) being able to complete a Dutch questionnaire, and (iii) giving written consent. All participants provided informed consent and filled out an extensive questionnaire about habits, diet, life style and socioeconomic position. Study designs and protocols were approved by the medical-ethical committee of University Hospital of Antwerp (FLEHS I newborns: A02-045-46-47, 3 July 2002; FLEHS I adolescents: A03 053, 7 July 2003; FLEHS I adults: A04-41, 10 May 2004; FLEHS II reference population: A08 09, 13 May 2008; FLEHS II region of Menen: A08 09 addendum, 7 December 2009). Sample handling was carried out in accordance with the laws of Belgium on biobanking. Human samples were registered in “Biobank@VITO”, Mol, Belgium; ID: BB190064 [20]. Names and addresses were only available to fieldworkers and the study physician.

A more detailed description of the recruitment and sampling strategy is published elsewhere [18,21,22].

### 2.3. Home-Produced Chicken Eggs

Between the 6 June and the 8 July 2013 chicken eggs and soil samples of the foraging area were sampled in 15 gardens of residents near the ICS of Menen. In a first step, participants of the 2010 HBM study who indicated to consume home-produced chicken eggs were invited to participate in this study. Eighty-nine letters of invitation were sent out, resulting in 10 signed informed consents. Additionally, the study was announced via different communication media in the communities in the study area, such as local newspapers and the community website. Volunteers were selected to participate when they met the following inclusion criteria: (i) chickens were kept by private individuals without commercial interests, (ii) the chickens were producing eggs, (iii) the chickens were free-range. This resulted in 15 selected locations: 3 locations in the neighboring community of Wervik, and 12 locations in the 2010 HBM study area, of which 7 were within and 5 outside the precautionary area. Each participant was asked to collect 10–15 eggs. At one location, an insufficient number of eggs were available, so finally analyses were performed on eggs from 14 locations. The eggs were stored at 4 °C until collection by the fieldworker.

Following several incidents regarding the food chain during the 1990s, a European strategy was developed to reduce human exposure to PCDD/Fs and PCBs, including establishing standards in food and feed on the market [16]. When home-produced eggs are not made available on the commercial market, official maximum levels of contaminants for commercially produced chicken eggs do not apply. Therefore, safe concentrations were calculated as the maximum levels that can occur in home-produced chicken eggs for safe consumption, based on toxicological guidance values for intake of PCDD/Fs, DL-PCBs, marker-PCBs and dichlorodiphenyltrichloroethane (DDT) and dichlorodiphenyldichloroethylene (DDE). The sum of PCDD/Fs and DL-PCBs has been shown to be the critical parameter for defining the consumption recommendations. By comparing observed POP levels in home-produced eggs with these calculated safe values, dietary recommendations for safe consumption were prepared. More information about the process of derivation is published separately [23].

### 2.4. Selection of Biomarkers

In the FLEHS I and II HBM studies, a selection of biomarkers of exposure were measured, starting with nine biomarkers of exposure in FLEHS I and elaborated to more than 40 in FLEHS II [19]. In this article, we focus on the results for dioxin-like compounds and PCBs, as these were the initial reason for concern in the region of Menen. In the HBM studies dioxin-like activity was assessed using the CALUX-assay (Chemically Activated Luciferase gene expression, see Section 2.6). The main argument for using the CALUX assay over high-resolution gas chromatography coupled with mass spectrometry (HR GC-MS) to assess internal levels of dioxin-like compounds was the low volume of serum needed at the time the studies were conducted (2–5 mL for the CALUX assay compared to 20 mL for the HR GC-MS method). A comparison of both methods based on 47 pooled samples showed comparable tendencies in the relative regional differences for both methods, although the absolute levels were not comparable [24] (see also Section 1 of the Supplementary Materials and Figure S1). The sum of PCB 138, 153 and 180 was used as a biomarker for exposure to PCBs, since the sum of these congeners multiplied by a factor of two is a good representation of the total PCB internal exposure [25]. In FLEHS I, the p,p′-DDE results were also identified as a persistent organic pollutant of importance for exposure in the region of Menen and are therefore included in this article. DDE is a degradation product of the frequently used organochlorine pesticide DDT, associated with adverse health effects. Internal levels of p,p′-DDE are considered to be a good biomarker for exposure to DDT compounds [26]. In Belgium, the use of DDT was banned in 1976.

Based on the FLEHS II results, possible contamination of home-produced eggs with dioxin-like compounds and DDT compounds was assessed by measuring 17 PCDDs (2,3,7,8-tetrachlorodibenzo-p-dioxin or 2,3,7,8-T4CDD, 1,2,3,7,8-pentachlorodibenzo-p-dioxin or 1,2,3,7,8-P5CDD, 1,2,3,4,7,8-hexachlorodibenzo-p-dioxin or 1,2,3,4,7,8-H6CDD, 1,2,3,6,7,8-hexachlorodibenzo-p-dioxin or 1,2,3,6,7,8-H6CDD, 1,2,3,7,8,9-hexachlorodibenzo-p-dioxin or 1,2,3,7,8,9-H6CDD, 1,2,3,4,6,7,8-heptachlorodibenzo-p-dioxin or 1,2,3,4,6,7,8-H7CDD, octachlorodibenzo-p-dioxin or O8CDD, 2,3,7,8-tetrachlorodibenzofuran or 2,3,7,8-T4CDF, 1,2,3,7,8-pentachlorodibenzofuran or 1,2,3,7,8-P5CDF, 2,3,4,7,8-pentachlorodibenzofuran or 2,3,4,7,8-P5CDF, 1,2,3,4,7,8-hexachlorodibenzofuran or 1,2,3,4,7,8-H6CDF, 1,2,3,6,7,8-hexachlorodibenzofuran or 1,2,3,6,7,8-H6CDF, 1,2,3,7,8,9- hexachlorodibenzofuran or 1,2,3,7,8,9-H6CDF, 2,3,4,6,7,8- hexachlorodibenzofuran or 2,3,4,6,7,8-H6CDF, 1,2,3,4,6,7,8-heptadibenzofuran or 1,2,3,4,6,7,8-H7CDF, 1,2,3,4,7,8,9-heptadibenzofuran or 1,2,3,4,7,8,9-H7CDF, octadibenzofuran or O8CDF), 12 DL-PCBs (non-ortho PCBs: PCB 77, 81, 126, 169, mono-ortho PCBs: PCB 105, 114, 118, 123, 156, 157, 167 and 189), six marker PCBs (PCB 28, 52, 101, 138, 153 and 180), DDT isomers (p,p′-DDT, o,p’-DDT), DDE isomers (p,p′-DDE, o,p’-DDE) and dichlorodiphenyldichloroethane (DDD) isomers (p,p′-DDD, o,p’-DDD).

### 2.5. Chemical Analysis

Serum and cord blood PCBs (PCB 138, 153, 180) and p,p′-DDE were analyzed using optimized and validated protocols [27]. In short, PCBs and p,p′-DDE were extracted from the serum and cord blood samples by solid-phase extraction on OASIS HLB cartridges and eluted with 10 mL dichloromethane. The extracts were concentrated and purified on 0.5 g of acidified silicagel (44% sulfuric acid (H_2_SO_4_), *w*/*w*), followed by elution with n-hexane. Further analysis was performed with gas chromatography for separation from other compounds, coupled with mass spectrometry detection with electron impact ionization. The limit of quantification (LOQ) for these compounds was 0.02 µg/L. Dioxin-like activity in cord blood was assessed by BDR-CALUX® (Chemically Activated Luciferase gene expression) (LOQ = 0.018 pg CALUX-TEQ/well), reflecting both PCDD/Fs and DL-PCBs [28,29]. After denaturation of proteins in cord blood with isopropanol, DL-compounds were extracted with n-hexane. The extracts were purified on a silica column with H_2_SO_4_. The eluate was evaporated and transferred in 7.5 μL dimethyl sulfoxide (DMSO). This extract was diluted 1/100 in medium and dosed to H4IIE rat hepatoma cells for 24 h, together with 2,3,7,8-TCDD standards in triplicate in a 96-well culture plate. The BDR-CALUX assay cell line was transfected with an aryl hydrocarbon receptor (AhR) -controlled luciferase reporter gene construct. Binding of dioxin-like compounds to the receptor results in production of luciferase, which can be measured with a luminometer. The response of the TCDD standard was used as a reference to convert the response of the sample into bioassay toxic equivalents (BEQ). In serum from adolescents and adults the dioxin-like activity was assessed by XDS-CALUX®, with separate assessment for PCDD/Fs and DL-PCBs [30]. Following this protocol, DL-compounds were first extracted from the serum samples with acetone (20 mL) and hexane (3 × 5 mL). Proteins were removed by a celite column and blood lipids collected by elution with hexane. The extract was further purified by an acidified silica column, connected in series with a carbon column. The PCB fraction was eluted with 15 mL hexane/ethyl acetate/toluene (8/1/1) and the dioxin fraction with 20 mL toluene. The purified extracts were transferred into 4 µL DMSO and dosed to the H1L7.5c1 mouse cell line. Each cell plate also included ten 2,3,7,8-TCDD standards, three quality control standards and three DMSO blanks. Dioxin-like activity was then measured using the AhR-controlled luciferase reporter gene as described for the BDR-CALUX assay. The measured luminescence of the samples was converted into bioassay toxic equivalents by comparison with the 10-point dose-response curve of the TCDD standards. The limit of quantification (LOQ) was 0.05 pg BEQ/g serum for PCDD/F activity and 0.03 pg BEQ/g serum for DL-PCBs activity, or 0.1 pg CALUX-TEQ/well. The plasma total lipid concentration (TL) was calculated from levels of triglycerides (TG) and total cholesterol (CHOL) [31], using Equation (1).

TL = (1.12 * CHOL) + (1.33 * TG) + 148(1)

where CHOL and TG are expressed in mg/dL.

The chicken eggs collected at each location were boiled for 10 min and pooled together. After cooling, the yolk was separated from the egg white. The yolks were mixed with sodium sulfate and extracted via Soxhlet with hexane: acetone (50:50). The fat content was subsequently determined gravimetrically. The fat extracts were then spiked. The samples were purified on a multi-layer column and an alumina column. Matrix interferences were removed, and PCDD/Fs and PCBs were collected in different fractions. The levels of PCDDs, PCDFs, dioxin-like PCBs and marker PCBs were determined with high-resolution mass spectrometry (GC-HRMS) in combination with the isotope dilution technique. At various stages, 13-C labeled 2,3,7,8-chloro-subsituted congeners were added to correct for any losses. The levels of DDT, DDE and DDD were determined via gas chromatography electron capture detection (GC-ECD). LOQ values for all measured congeners are given in the Supplementary Materials (Supplementary Table S5 in Section 2).

### 2.6. Statistics

#### 2.6.1. HBM Studies

Database management and statistical analysis were performed using SAS for Windows (SAS Institute Inc., Cary, NC, USA), version 9.1 in FLEHS I and 9.2 in FLEHS II and Statistica, version 7.1 in FLEHS I. For biomarkers of exposure, geometric means with 95% confidence intervals (95% CI) were calculated on medium-bound concentrations, setting values below the limit of quantification (LOQ) on half of the LOQ. The reference values in FLEHS I were calculated as the mean value of all pooled study areas, with the contribution of each area weighted proportional to the number of inhabitants, and adjusted for age, sex and smoking in regression analysis [18,28]. Reference values for FLEHS II were calculated on raw data from the reference population of Flanders. Analysis of variance (ANOVA) was used to study differences in biomarker levels between the study population in the ICS region and the Flemish reference values. Comparison of proportions in study population characteristics between both study populations in FLEHS II (region of Menen and Flemish reference population) was performed using the chi-square test. Statistical significance is considered at the 5% level. Associations between possible covariates and biomarker levels were tested using multiple linear regression analysis. To achieve normal distribution, biomarker levels were transformed using the natural logarithm. These transformed variables were used as response variables or dependent variables in the regression analysis. Assumptions of normality, constancy of variance (homoscedasticity), independence (randomness) and multicollinearity were checked with informal plots (normality and constancy of variance) and formal tests (Durbin-Watson test for constancy of variance and variance inflation factors for multicollinearity) and were fulfilled for the final models. All independent variables were categorical, implying testing for linearity was not needed. The multiple linear regression analysis was performed using Equation (2):

E(Y_x_) = α + β_1 × 1_ + β_2 × 2_ + β_3 × 3_ (2)

where Y_x_ is the predicted value of the response variable, α the Y-intercept, X_1_, X_2_ and X_3_ the tested covariates, and β_1_, β_2_, and β_3_ the effects of X_1_, X_2_ and X_3_ respectively on Y. Since the dependent variables were transformed using the natural logarithm, the resulting beta coefficients (β) in the regression models are estimated for the transformed response variables. To interpret the regression results on the original scale of the dependent variables, the estimates were transformed using the exponent function (e^β^), meaning that when covariate X increases with one unit (X + 1), the dependent variable Y increases with a factor e^β^ (Y * e^β^). A value of e^β^ equal to one, means no change in Y, when e^β^ equals 1.50 this means a 50% increase of Y in with each unit increase of X.

For POPs, blood lipid content, age, sex, body mass index (BMI) and smoking status were forced in the models. Other covariates were retained in the models at *p* < 0.05. More information on the statistical methodology is available elsewhere [18,28,32].

#### 2.6.2. Home-Produced Chicken Eggs

Descriptive statistics (median, 10th and 90th percentiles) of PCDD/Fs, DL-PCBs, marker PCBs, DDT, DDE and DDD in eggs were calculated using Statistica 11 software. For the descriptive statistics values below the LOQ were set on half the LOQ. For PCDD/Fs and DL-PCBs levels were also converted to concentrations expressed in toxic equivalents (TEQ) by multiplying levels in pg/g lipid weight for each congener by corresponding toxic equivalent factors (TEF) according to WHO 1998 [33] and WHO 2005 [34]. For comparison of levels in the eggs, samples from each location with the calculated safe concentrations for home-produced eggs the LOQ were used for values below LOQ, representing the worst-case scenario.

### 2.7. Participation of the Local Community

The local community played an important role in the development of the case study, as described in this article. Representatives of the local community initially consisted of a group of citizens concerned about local pollution, as well as the local authorities of the city of Menen and the surrounding municipalities. Already before the first HBM study, these representatives were meeting on a regular basis, within a technical working group of the local environment council of Menen, to closely monitor and discuss evolutions in environmental measurement data and related topics. This working group was also attended by local services of the regional government (such as the Flanders Environment Agency and the environmental and health inspectorate), as well as representatives of several of the companies. Members of this working group managed to bring the topic to the attention of policy makers and to attract research to the region, such as the FLEHS studies.

The final decision to include the MWI of Menen in FLEHS I was made by the Flemish government. The decision to include the region of Menen in FLEHS II was made in the context of a structured and transparent selection procedure, in which societal concern was one of the selection criteria [35].

From the start of the FLEHS II study, the local technical working group was expanded to include delegates of the local schools and general practitioners, to act as a local advisory board. Between December 2009 and November 2011, five meetings were organized. Scientists of the research consortia prepared the study design, among which the choice of biomarkers, biological media, chemical and statistical methods, and presented their choices and results to the local advisory board for feedback. The local advisory board assisted in recruiting participants and provided advice on the study design (delineation of the study area, information on local sources and industrial activities) and communication. Local meetings were regularly attended by scientists involved in the research to facilitate dialogue between the academic experts, the local community and policy makers.

After the FLEHS II study, the local advisory board was maintained, and was involved in discussions on the interpretation and policy-uptake of the HBM results. To this end, a participation process was initiated by the Flemish government, involving scientists, policy makers and local stakeholders. The goal of this process was to achieve a joint interpretation of the research results and to develop an action plan that was both evidence-informed and locally supported. Following the principles of an analytical deliberative approach, phases of desk research and expert consultation were alternated with local deliberation, recognizing the importance of both academic and local knowledge [36]. This approach in the context of the FLEHS studies was previously described [37,38]. In addition, a focus group with citizens was organized to further explore support for policy options. For this focus group, the adolescents who participated in the FLEHS II study were invited, as well as their parents.

In the context of the study on chicken eggs, members of the local advisory board were invited to engage in the steering committee, together with the commissioning authorities. Between February 2013 and March 2014, five meetings were organized. Local stakeholders had input into the study design and actively participated in promoting the study and recruiting participants. The study results and dietary recommendations for safe consumption of home-produced chicken eggs were also discussed with the local advisory board.

These studies were financed by the Flemish government; the local advisory board did not contribute financially to the studies.

## 3. Results

### 3.1. HBM Studies

In the first HBM study (sampling period 2002–2005), mean levels of the sum of three marker PCBs in newborns and adolescents (14–15 years) living near the ICS of Menen were almost twice as high compared to the weighed reference mean of all eight types of area (Table 2). In newborns near the ICS of Menen, significantly higher p,p′-DDE values were also observed. In the adult study population (50–65 years), POPlevels in the Menen area were not significantly different from the overall mean values. Statistical analysis performed on the FLEHS I data of all eight types of area combined were published previously, and revealed that higher consumption rates of locally produced food were associated with higher internal levels of marker PCBs and p,p′-DDE [32]. Consumption of locally grown vegetables was associated with higher body burdens of p,p′-DDE in mothers of newborns (+18%, *p* < 0.01) and in adolescents (+13%, *p* < 0.05). Consumption of locally produced meat was associated with higher body burdens of p,p′-DDE (+19%, *p* < 0.001) in adolescents and of PCBs in adolescents (+16%, *p* < 0.001) and adults (+10%, *p* < 0.001). Consumption of locally produced dairy by mothers of newborns was associated with higher levels of PCBs (+11%, *p* < 0.05) in cord blood.

In the second HBM study (sampling period 2010–2011) the adolescents with residence in the region of Menen surprisingly showed significantly lower serum levels of marker PCBs, p,p′-DDE and dioxin-like activity (PCDD/F-CALUX and DL-PCB-CALUX) compared to levels in peers recruited all over Flanders (Table 2). Observed levels in the second study were also lower compared to the first study (2004) for PCBs (37 versus 114 ng/g lipid weight) and p,p′-DDE (48 versus. 117 ng/g lipid weight). CALUX results were not available for adolescents participating in the first HBM study. A summary of the characteristics of the FLEHS II study populations in the region of Menen and in Flanders are presented in Table 3. More details on the characteristics of both study populations are available in Table S1 in Section 2 of the Supplementary Materials.

Multiple linear regression models using the data of both the study population of Menen and the reference study population in Flanders (Table 4) showed that consumption of locally produced chicken eggs, locally produced vegetables and locally caught fish were associated with higher serum levels of marker PCBs (+19.2%, *p* = 0.003; +12.1%, *p* = 0.046 and +26.8%, *p* = 0.018, respectively) and of p,p′-DDE (+71.5%, *p* < 0.001; +40.1% *p* < 0.001 and +86.1%, *p* < 0.001, respectively). Study population characteristics showed significantly less consumption of locally produced food (chicken eggs, vegetables, fruit) by adolescents in the Menen region compared to their peers in Flanders (Table 3). Since both, the consumption of locally produced eggs and of locally produced vegetables, were associated with higher serum levels of marker PCBs and p,p′-DDE, the significantly lower consumption of these products by the adolescents in Menen in comparison with the reference population partly contributed to the lower observed body burdens of these POPs in the participants of Menen. Consumption of locally caught fish was also associated with higher serum levels of marker PCBs and p,p′-DDE. However, there was no statistical difference in consumption of locally caught fish between the participants in Menen and in the Flemish reference group. Therefore, it is less likely that consumption of locally caught fish contributed to the observed differences in body burdens of these POPs between both study populations. More details on the multiple linear regression models are available in the Supplementary Materials (Supplementary Table S2 in section 2).

**Figure 3:**
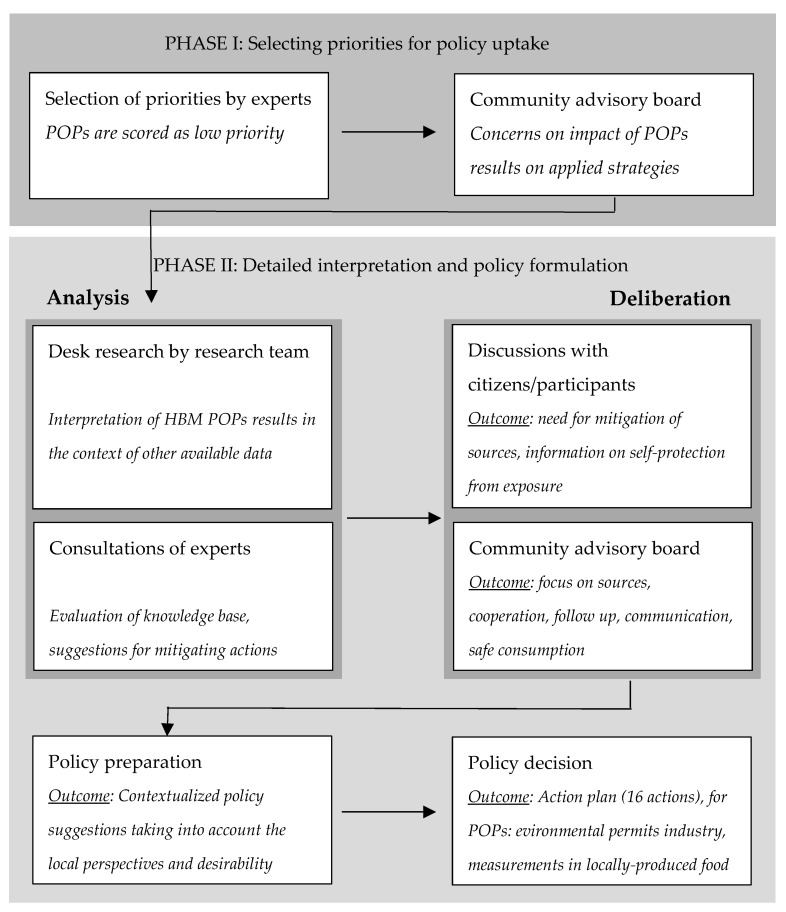
Overview of the successive steps in the policy uptake of the FLEHS II human biomonitoring results in the region of Menen. POPs: persistent organic pollutants.

Figure 3 presents a schematic overview of the structured and participatory process for the joint interpretation and policy uptake of the FLEHS II HBM results in the ICS region of Menen.

In addition to the POPs measurements, a range of other biomarkers was measured in the FLEHS II study in Menen (outside the scope of this article). A first objective of the participation process that started after FLEHS II, was therefore to determine the priorities for further policy development. In first instance, the POPs results were not identified as a high priority by consulted experts (Supplementary Table S3 in section 2 of the Supplementary Materials), given the favorable results compared to the Flemish reference group and decreasing time trends. However, after discussions with the local advisory board, it soon became clear that the local community’s concerns had not subsided, and the question arose as to whether the precautionary area could be abolished. Because of this, the POPs results were picked up in the science-to-policy process.

In a next phase, the selected results were analyzed in more detail, additional information was summarized (including most recent environmental data) and experts were consulted to evaluate the knowledge base and the need for policy actions, involving both academic experts and local experts (such as the environment department of the city of Menen, the Flanders Environment Agency and experts from local companies). Suggested actions included an intensified follow up and mitigation of local (industrial) sources, additional measurements in environmental media, awareness raising strategies on safe consumption of locally produced food, better access to information and transparent decision making. Discussions with a focus group of participants of the HBM study and with the local advisory board captured the perspectives of local residents and the local desirability of the various suggested actions (Supplementary Figure S2 in section 2 of the Supplementary Materials).

The participatory process resulted in a tailored policy action plan for the region of Menen, consisting of 16 different actions (see Section 3 of the Supplementary Materials), established by the Flemish government (departments of environment and of public health) and the local authorities. Concerning POPs, the action plan included intensified attention to environmental permits of local companies, as well as additional measurements of PCDD/Fs, DL-PCBs and DDT-compounds in soil samples of gardens and in home-produced chicken eggs of locations in Menen and neighboring communities Wervik and Wevelgem, with public communication of the study results.

### 3.2. Deriving Dietary Advice on Safe Consumption of Home-Produced Chicken Eggs

This study on home-produced eggs was conducted to achieve one of the actions mentioned in the local action plan that was derived from the participatory interpretation of the FLEHS II HBM results. Descriptive characteristics of the coops at the 15 locations in the study area are presented in Table S4 of the Supplementary Materials. The majority of the participants owned up to 6 chickens, held in a coop of 15 m^2^ or less. In 73% of these locations less than a quarter of the foraging area was covered with vegetation. Vegetation cover limits ingestion of soil particles by the chickens. At all locations, chickens were given table leftovers, and in 80% of the locations, weeds were thrown into the coop area. Soil particles that stick to the weeds’ roots can also be ingested by the chickens. At four locations, insecticides (not specified) were used in the coop, and at none of the locations were burning ashes of stoves used in the coop.

Descriptive statistics of the concentrations of PCDD/Fs, DL-PCBs, marker PCBs and DDT-compounds in home-produced chicken eggs from 14 locations in the Menen region are given in section 2 of the Supplementary Material (Supplementary Tables S5–S7). Comparing the observed levels in the collected eggs with the calculated values for safe consumption [23] resulted in scientific dietary recommendations for residents in the region of Menen (Table 5), being a maximum of one egg per month for children under 6 years of age, one egg every two weeks for children between 6 and 12 years old, and one egg a week for citizens 12 years and older. This would imply an adjustment of the previous local advice for the precautionary area, which was more stringent.

This scientific advice was discussed with the local advisory board, taking into account the results from the HBM studies and environmental monitoring. Continued deposition measurements of the Flanders Environment Agency and the AEROPA project in 2012 still showed repeated exceedances of the monthly average threshold value for PCDD/Fs and DL-PCBs at two locations in the residential areas near the ICS [39]. Based on the combined weight of evidence and to ensure further beneficial results in POP body burdens, the local advisory board recommended adhering to the precautionary principle and maintaining the advice not to consume locally produced eggs in the precautionary area. The responsible Flemish and local authorities adopted the recommendations of the advisory board, which were communicated in a leaflet for the citizens of Menen and two neighboring communities (Table 6). Within the precautionary area all consumption of home-produced eggs remained discouraged. Outside this precautionary area, consumption was restricted to a maximum of one egg every two weeks for children under 6 years of age, one egg a week for children between 6 and 12 years old, and two eggs a week for citizens 12 years and older. For consumption of commercial eggs, the Belgian dietary recommendations should be applied (Table 6), which include eggs used in the preparation of food [40]. Meanwhile, the Belgian dietary recommendations changed to double the initial recommendations at the time of the study.

## 4. Discussion

The main objective of this article was to illustrate, by means of a case study, how HBM studies can contribute to evidence-informed policy. According to Bowers and Testa [41], good evidence-informed policy making should involve cross-sectoral collaborations, use state-of-the-art research designs and contextualized knowledge, and should focus not only on exploring explanations and answers to causal questions, but also on what will work in a given context. The FLEHS program, organized by the Center of Expertise on Environment and Health, has supported the policy decisions in the region of Menen by adding HBM expertise to the knowledge base and by stimulating the local dialogue between the community and local and regional policy makers, resulting in answering to local concerns. The pollution near MWIs has raised concern in other European countries as well. For example, in Italy [42], France [43,44] and Spain [45,46], human biomonitoring studies have been conducted to monitor the body burdens of PCDD/Fs and PCBs in subjects working or living in the surroundings of the MWIs. European HBM studies near metal recycling plant often focus on exposure to metals [47,48], but in a German HBM study, the body burdens of PCBs were monitored near a transformer recycling plant [49,50]. Here we discuss the aspects of our study contributing to the evidence-informed policy making.

### 4.1. HBM to Confirm High POPs Exposure Detected in Environmental Media

The first HBM FLEHS program (2002–2006) tested the hypothesis that differences in environmental pressure in Flanders are reflected in different body burdens of pollutants, indicating the importance of regionally and/or locally tailored policies [19]. The area near the MWI of Menen drew attention as higher levels of PCBs and p,p′-DDE were measured in blood samples from newborns and adolescents living near the MWI of Menen in comparison with the Flemish reference values of FLEHS I. These higher internal human pollution loads were in line with the higher levels in environmental media that had been reported previously and raised public concern in the area. Additionally, the FLEHS I results identified exposure routes, showing that consumption of locally produced food was significantly associated with higher body burdens of POPs [32]. These findings supplemented the earlier modelled dietary exposure calculations with the Xtrafood model [15], indicating home-produced chicken eggs to be major contributors to the PCDD/F exposure of residents. The decision in 2003 of local and Flemish authorities to install a precautionary area within the territory of Menen, in which consumption of locally grown food was discouraged, was thereby reaffirmed by the FLEHS I results. The importance of locally produced food consumption to body burdens of PCDD/Fs of residents living near MWIs was also reported in a French HBM study, with higher PCDD/F serum levels being observed in participants consuming locally produced food of animal origin [43].

### 4.2. HBM to Follow Up Exposure over Time and Evaluate Remediating Actions

The second FLEHS HBM study made it possible to follow up local exposure trends and broadened the scope to other environmental pollutants and health effects. Compared to the first HBM study, lower serum levels of PCBs and p,p′-DDE were observed in the Flemish reference adolescent population in general [51]. Furthermore, even lower levels were measured in adolescents residing in the Menen area, while deposition samples for the time period 2002–2012 analyzed by the Flanders Environment Agency [52], still showed elevated levels of PCB 126 in the Menen area. These favorable HBM results were associated with lower consumption of locally produced food by participants in the Menen area at the time of the second FLEHS study. These results suggest the positive impact of earlier local initiatives to inform residents of the Menen area about local pollution and to raise awareness about the contribution of locally produced food consumption to human exposure.

Similar observations have been reported for an urban area near a chemical factory in Northern Italy [53]. High levels of PCDD/Fs and PCBs in locally produced vegetables and animal products resulted in public health interventions, adopted in 2002 in the contaminated area. These consisted of a ban on all farming activities and fruit/vegetable cultivation in private gardens and limited access to public parcs. Two independent HBM studies, in 2003 and in 2013, showed decreasing PCB serum levels over time and consumption of locally produced food mainly occurring before 2002 [53]. According to the authors’ conclusions, the public health interventions, adopted in 2002, interrupted the food chain route of exposure and contributed to decreasing PCB serum levels. Interventions in dietary habits with respect to contaminated locally produced food were shown to be efficient in reducing exposure. However, discussions with local stakeholders and residents during the process of policy uptake of the FLEHS II HBM results indicated that source-oriented mitigating strategies were perceived to be highly desirable.

Significantly lower serum levels were also observed for PCDD/F-CALUX and DL-PCBs-CALUX in adolescents of the Menen study population compared to the Flemish reference group. For these biomarkers, it is less likely that a lower consumption of locally produced food in the area of Menen in comparison to the Flemish reference group contributed to the observed difference in serum level, since no significant associations between the serum levels and consumption of locally produced food could be demonstrated. A lack of association between consumption of locally produced food and serum levels of PCDD/Fs was also observed in a Spanish HBM study near an MWI in Bilbao [46]. Emissions of MWIs were restricted to 0.1 ng TEQ Nm^−3^ in 2000, and the MWI in Menen was closed in 2005. Yet, on the French side of the border, an MWI is still active. Decreasing time trends of emissions and environmental levels of PCDD/Fs and PCBs in the Menen area were observed [52]. Other HBM studies near MWIs in Europe, conducted after the introduction of emission restrictions in 2001, concluded that living near MWIs no longer significantly contributed to the PCDD/F serum levels of nearby residents, indicating that measures taken to reduce the emissions of MWIs had been effective [42,43]. Additionally, in a residential area near an hazardous waste incinerator in Spain, declining serum levels of PCDD/Fs were reported from successive HBM studies conducted in 1998, 2002, 2007, 2012 and 2018 [45]. However, exceedances of the Flemish deposition guiding values for PCDD/Fs and PCB 126 still occurred in the region of Menen [13].Next to the MWIs in France, in the region of Menen a large metal recycling plant was still active at the time of the HBM studies. These activities and accidental fires at the metal recycling plan are possibly contributing to PCDD/F and PCB 126 depositions. High deposition levels of PCDD/Fs and PCBs have also been reported near other metal recycling plants in Belgium [54]. In Germany in 2010, six marker PCBs and 12 DL-PCBs were measured in the serum of workers in a transformer recycling plant, their relatives and of people working or living nearby the factory [49]. Compared to the general population, higher PCB serum levels were observed in (former) workers and their families, but not in residents living in the surroundings of the plant. That HBM study facilitated a health surveillance program as a response to increasing concern of workers, their families and local residents about their potential exposure and possible health effects [50].

In sum, these results show the importance of both emission restrictions and additional local initiatives to limit exposure, as well as the added value of HBM to follow-up exposure over time and to evaluate policy effectiveness. A limitation of HBM as a tool for exposure assessment is that these data hold no information on the sources the exposure originated from [55]. Therefore, combining body burden information from HBM data with additional environmental data and individual questionnaire data enhances opportunities for policy makers to make informed decisions.

### 4.3. HBM Embedded in a Participatory Approach to Enable Community Involvement and Support for Policy Actions

The different research activities in the region of Menen started in response to real concerns among the local community, and managed to address these concerns. Members of the local community followed the entire process and were actively involved in different study activities. In this way, local stakeholders were able to influence important decisions: first, the sustained attention for POPs in the region, even after the favorable results of FLEHS II, and second, the decision to maintain the precautionary area after new measurements in local eggs. The initial reason for the concern of local stakeholders about exposure of residents to PCDD/Fs and PCBs seemed to be resolved after the second HBM study. However, the participatory approach captured new concerns of citizens and local authorities regarding the consequence of these results for current measures, such as the advice not to consume locally produced food in the precautionary area. Placing the science-based dietary recommendations, based on new measurements, in the context of the lower POP body burdens being partly explained by lower consumption of locally produced food, and the continuing exceedance of the dioxin deposition guiding values near the ICS, resulted in the decision to maintain the advice not to consume locally produced food in the precautionary area. Transparency in the decision-making process and the involvement of local stakeholders added to the understanding and support for this measure among the local citizens. This case is therefore also a good example of community-based participatory research [56], as well as the added value of such an approach. Scientific information on exposure was complemented with contextual information. In-depth discussions with local stakeholders and citizens helped to understand the perspective of local residents and to take into account the local desirability of the various suggested policy actions. In this way, policy makers also focused on what would work given the local context, which resulted in a policy action plan supported by authorities, experts, societal stakeholders and industry with commitments of all parties involved.

The participatory approach, involving the local community, also experienced some challenges. This approach implies a certain flexibility in the study design. Consulting the local community at different stages during the study requires that sufficient time is allowed for this in the research planning and that changes to the study design can be made along the way. The attitude of local residents and stakeholders towards our studies was also shaped by the local context, the personal understanding of a complex topic and previous experiences, e.g., with previous local research projects. Different stakeholders involved often had different expectations and needs, not all of which were answered within the scope of the research projects. Nevertheless, by using a participatory approach in the region of Menen, the consortium and responsible authorities intended not only to contribute to the knowledge base concerning the local situation, but also to facilitate a constructive dialogue on environmental health between citizens, authorities, experts and companies, leading to a better (mutual) understanding of the local situation, an integration of different knowledge sources, and above all transparent decision making with respect to priorities, policy actions, and the results of those actions.

In a final remark, we would like to address the further evolution in this region, from after the study of home-produced eggs in 2013 up to the present situation in 2021. Deposition measurements of PCDD/Fs and PCB 126 near the ICS were continued by the Flanders Environment Agency. The Flemish deposition guidelines for these compounds are still regularly exceeded in the residential area near the ICS: two times in the period September 2017–November 2018 [57], three times in the period December 2018–October 2019 [58] and one time in the period November 2019–September 2020 [59]. These exceedances are probably a result of the diffuse emissions from the metal recycling plant and accidental fires on the site. Intensified inspections by the environmental inspectorate, as part of the action plan that resulted from the participatory process after FLEHS II, indicate that guided emissions from the metal recycling plant are far below the emission guidance values set in the environmental permit of the company. In October 2020, new legislation was implemented for metal recycling plants, based on the conclusions of the Best Available Techniques Reference document (BREF) on Waste treatment [60]. The local action plan also included actions to improve communication between local industry, local authorities, and citizens. To achieve a better mutual understanding among these parties, a participation project was initiated in 2014. Citizens, representatives of the local authorities, and local industry representatives meet regularly to discuss the emissions of the metal recycling plant and deposition results in the residential areas. In addition, citizens are informed via a website. On this website, citizens can find, for instance, the local action plan, the reports and results from studies, the advice on consumption of home-produced eggs, and a list of streets that are within the precautionary area.

## 5. Conclusions

In a residential area near an ICS with a (former) municipal waste incinerator and a large metal recovery plant, environmental data of PCDD/Fs and PCBs measured in air and in deposition, caused health concerns among residents and local and regional authorities. Targeted human biomonitoring (HBM) near the ICS provided accurate information on the real-life body burdens of environmental chemicals in local residents. HBM helped in filling the gaps between observed levels of pollutants in environmental samples and actual body burdens of citizens, using state-of the-art knowledge of biomarkers. In this way, HBM added to the weight of evidence and contributed to answering the question as to whether living near industrial activities was associated with increased internal chemical exposure. The combination of individual biomarker results and questionnaire data on food consumption identified locally produced food to be an exposure route of importance. This supported health promotion measures that discouraged the consumption of locally produced food in a delineated area. Repeating the HBM study made it possible to follow up local exposure trends and to evaluate the combined effect of exposure reduction strategies. However, since HBM aggregates exposure from different sources, HBM data were complemented with environmental data, indicating local industrial sources as possible contributors to the observed body burdens in residents. The participatory approach used in the policy uptake of the HBM results created transparency and societal support for the application of mitigating actions and a more contextualized policy action plan. For POPs, this action plan resulted in deriving specific recommendations on the safe consumption of home-produced chicken eggs in the region of the case study.

## Figures and Tables

**Table 1:** Number of participants in each study population of FLEHS I and FLEHS II (Flemish Environment and Health Study), for the reference group as well as the region near the industrially contaminates site (ICS) in Menen.

Study Population	FLEHS I (2002–2006)	FLEHS II (2007–2011)
Reference group	Sum 8 types of areas	Representative for Flanders
newborns	*n* = 1583	*n* = 255
Adolescents (14–15 years)	*n* = 1679	*n* = 210
Adults (20–40 years)		*n* = 204
Adults (50–65 years)	*n* = 1583	
Region ICS Menen	Part of the 8 types or areas	Additional study population
newborns	*n* = 14	
Adolescents (14–15 years)	*n* = 14	*n* = 199
Adults (50–65 years)	*n* = 35	

**Table 2:** Comparison of the geometric mean concentrations (95% confidence interval) of dioxin-like compounds, marker PCBs and p,p′-DDE in newborns, 14–15 year-old adolescents and 50–65 year-old adults residing near the ICS of Menen with the reference mean values of all eight areas combined (FLEHS I 2002–2006) and the Flemish reference mean (FLEHS II 2007–2011). FLEHS I results adjusted for age, sex and smoking, FLEHS II results are raw data.

	FLEHS I Newborns (2002–2004)		FLEHS I Adolescents (2003–2004)		FLEHS I Adults (2004–2005)		FLEHS II Adolescents (2007–2011)	
	Mean of 8 Areas	MWI Menen+	Mean of 8 Areas	MWI Menen	Mean of 8 Areas	MWI Menen	Flemish Reference Group	ICS Menen
N	1196	25 ^1^	1679	14	1583	35	210	199
Dioxin-like compounds (CALUX)								
pg CALUX-TEQ/g lipid weight	22.6 (21.4–23.8)	25.4 (17.5–37.0)			19.2 (18.2–20.2)	16.9 (14.6–19.1)		
PCDD/Fs (CALUX)								
pg BEQ/g lipid weight							110 (104–116)	70.0 ** (65.5–74.9)
Dioxin-like PCBs (CALUX)								
pg BEQ/g lipid weight							32.7 (30.7–34.7)	29.1 * (27.5–30.8)
Sum PCBs 138 + 153 + 180								
ng/g lipid weight	64.4 (61.1–67.9)	111 ** (79.8–154)	67.6 (65.6–69.6)	114 ** (112–116)	333 (325–341)	373 (371–375)	49.6 (45.7–53.8)	37.2 ** (34.1–40.6)
p,p′-DDE								
ng/g lipid weight	110 (104–116)	181 * (126–259)	94.3 (89.3–99.5)	117 (114–119)	423 (398–449)	425 (423–428)	70.8 (63.6–78.8)	47.9 ** (43.2–53.0)

^1^ number of participants in 7 municipalities with MWIs of which 14 participants were recruited in Menen; * *p* < 0.05, ** *p* < 0.001. BEQ: bioassay toxic equivalents; CALUX: Chemically Activated Luciferase gene expression; p;p’-DDE: dichlorodiphenyldichloroethylene; MWI: municipal waste incinerator; PCBs: polychlorinated biphenyls; PCDD/Fs: polychlorinated dibenzo-p-dioxins and furans; TEQ: toxic equivalents.

**Table 3:** Characteristics of the study population near the Menen ICS and of the reference group of FLEHS II (2010–2011).

Characteristics	Flemish Reference Group (*n* = 210)	ICS Menen (*n* = 199)	*p*-Value
Consumption of locally produced vegetables			<0.01
yes	39.6%	22.2%	
Consumption of locally produced fruit			<0.01
yes	23.3%	4.1%	
Consumption of locally produced chicken eggs			<0.01
yes	45.0%	19.3%	
Consumption of locally caught fish			0.57
yes	8.5%	7.0%	

**Table 4:** Multiple linear regression results on consumption of locally produced food (% change compared to reference category and 95% CI) for PCDD/F-CALUX, dioxin-like PCBs-CALUX, sum 3 PCBs (138 + 154 + 180) and p,p′-DDE in serum (ng/L) for 14–15 year-old adolescents of the FLEHS II study population in the ICS Menen and the Flemish reference group combined (*n* = 409).

Covariates	PCDD/F-CALUX ^1^ % Change (95% CI)	DL-PCB-CALUX ^1^ % Change (95% CI)	Sum 3 PCBs ^2^ % Change (95% CI)	p,p′-DDE ^3^ % Change (95% CI)
Consumption of locally produced eggs				
Yes vs. no	−5.4 (−14.3; +4.4)	+3.4 (−5.5; +13.2)	+19.2 (+6.2; +33.8) **	+71.5 (+46.7; +100.4) ***
Consumption of locally produced vegetables				
Yes vs. no	+1.4 (−8.1; +11.7)	−1.3 (−9.6; +7.8)	+12.1 (+0.2; +25.4) **	+40.1 (+19.9; +63.8) ***
Consumption of locally produced fruit				
Yes vs. no	+6.8 (−6.8; +22.4)	+11.4 (−1.4; +26.0)	+11.8 (+0.9; +30.6)	+19.0 (−4.3; +47.9)
Consumption of locally caught fish				
Yes vs. no	−1.2 (−17.0; 16.0)	−4.1 (−17.6; +11.6)	+26.8 (+4.2; +54.3) *	+86.1 (+41.1; +145.5) ***

^1^ adjusted for blood lipids, sex, age, BMI, smoking, area of residence; ^2^ adjusted for blood lipids, sex, age, BMI, smoking, breastfed as a baby, school type of the participant, season, area of residence; ^3^ adjusted for blood lipids, sex, age, BMI, smoking, breastfed as a baby, area of residence; * *p* < 0.05; ** *p* < 0.01; *** *p* < 0.001.

**Table 5:** Calculated values for safe consumption of home-produced eggs, with corresponding dietary recommendations and number of measurement locations in each category.

Safe Value for Home-Produced Eggs	Dietary Recommendations			Number of Locations	
PCDD/Fs + DL-PCBs (pg TEQ_WHO98_/g Lipids)	Children < 6 y	6 y < Children < 12 y	Children > 12 y + Adults	Within Precautionary Area	Outside Precautionary Area
<7.8	2 eggs/week	4 eggs/week	6 eggs/week	1	1
7.8–15.0	1 egg/week	2 eggs/week	3 eggs/week	3	4
15.0–23.0	1 egg/2 weeks	1 egg/week	2 eggs/week	2	1
23.0–46.0	1 egg/month	1 egg/2 weeks	1 egg/week	1	1

**Table 6:** Policy recommendations on safe consumption of home-produced eggs in the Menen region, translated from the Dutch leaflet for citizens.

Age	I Prefer Home-Produced Eggs		I Prefer Commercial Eggs
	I Live within the Precautionary Area	I Live Outside the Precautionary Area	Belgian Dietary Recommendations ^1^
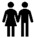 < 6 years	Do not consume home-produced chicken eggs	Max. 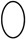 /14 days	Max. 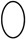 /week
6 years < 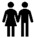 < 11 years	Do not consume home-produced chicken eggs	Max. 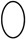 /week	Max. 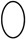 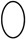 /week
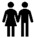 > 12 years	Do not consume home-produced chicken eggs	Max. 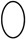 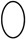 /week	Max. 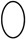 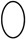 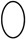 /week

^1^ current recommendations during the course of the study (2012) [40].

## Data Availability

The datasets used and analyzed in this article are available on request from the corresponding author. The data are not publicly available due to privacy and ethical aspect.

## Supplementary Materials

The following are available online at https://www.mdpi.com/article/10.3390/ijerph18115559/s1, Section 1: Additional information on the CALUX assay, Figure S1: Correlation between the CALUX (Chemically Activated Luciferase gene expression) results and the summed concentrations of polychlorinated dibenzo-p-dioxins and furans (PCDDs, PCDFs) and non-ortho polychlorinated biphenyls (PCBs) for 47 pooled serum samples. Section 2: Additional tables and figures; Table S1: Characteristics study population of the Menen ICS and the reference group of FLEHS II (2010–2011), Table S2: Multiple linear regression results (% change compared to reference category and 95% CI) for PCDD/F-CALUX, dioxin-like PCBs-CALUX, sum 3 PCBs (138 + 154 + 180) and p,p′-DDE in serum (ng/L) for 14–15 year-old adolescents of the FLEHS II study population in the ICS Menen and the Flemish reference group combined (n = 409), Table S3: Results prioritization of HBM results in the Menen region, Figure S2: Appreciation of the experts’ suggestions by the focus group with participants and their parents, Table S4: Descriptive characteristics concerning the coops at the 15 locations in the study area in the region of Menen, Table S5: Descriptive statistics for PCDD/Fs, DL-PCBs, marker PCBs and DDT compounds in home-produced eggs from the Menen region, Table S6: Descriptive statistics for PCDD/Fs, DL-PCBs in home-produced eggs from the Menen region, expressed in pg TEQ_WHO1998_/g lipid weight, Table S7: Descriptive statistics for PCDD/Fs, DL-PCBs in home-produced eggs from the Menen region, expressed in pg TEQ_WHO2005_/g lipid weight, Section 3: The local action plan for the region of Menen.
