# Supplementary material for: Human Biomonitoring Data Enables Evidence-Informed Policy to Reduce Internal Exposure to Persistent Organic Compounds: A Case Study
Source: Int J Environ Res Public Health. 2021 May 22;18(11):5559. doi: 10.3390/ijerph18115559 (PMC8196998; doi:10.3390/ijerph18115559)
Supplement: Supplementary file 1 [file ijerph-18-05559-s001.zip › ijerph-1189858-supplementary.pdf]

Supplementary material

Section 1: Additional information on the CALUX assay

Polychlorinated dibenzo-p-dioxins and furans (PCDD/Fs) are more accurately assessed using high resolution gas chromatography coupled with mass-spectrometry (HR GC-MS), compared to the CALUX (Chemically Activated Luciferase gene expression) assay. However the main argument in favor of using the CALUX assay in our human biomonitoring (HBM) study was the low volume of serum needed. For the CALUX assay 2-5 mL serum was used, whereas for the HR-method (in the time that the analyses were done), 20 mL of serum was needed. In the same line, the price of the chemical analysis was 4-fold higher. Especially the volume of blood needed was a crucial factor, as the analysis made part of a larger HBM study, in which blood also for assessment of other compounds was needed.

Considering the CALUX assay performance in comparison with HR-GCMS, a comparison was made between the measurements on 47 pooled serum samples (Koppen et al. 2001, https://pubmed.ncbi.nlm.nih.gov/11514106/) (see figure showing pooled serum samples analysed with both methods). It was observed that the absolute levels were not comparable, but the relative regional differences showed the same tendencies. This was considered sufficient for relative comparisons of the blood samples collected in different areas.

The laboratory did not participate in the EU proficiency test for halogenated POPs in Feed and Food, but was involved in the European project DIFFERENCE (Dioxins in Food and Feed – Reference methods and New Certified Reference Materials) (see: https://www.sciencedirect.com/science/article/abs/pii/S0165993606000483).

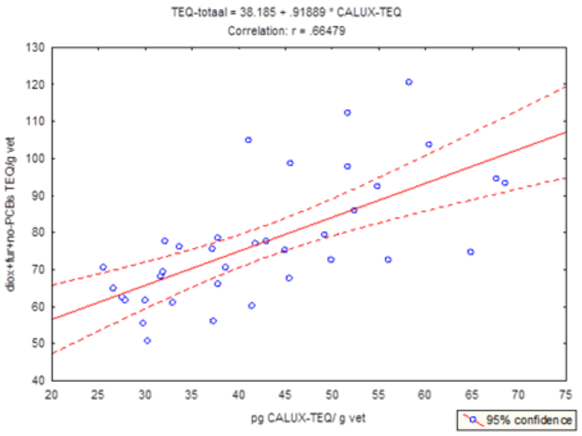

Figure S1. Correlation between the CALUX (Chemically Activated Luciferase gene expression) results and the summed concentrations of polychlorinated dibenzo-p-dioxins and furans (PCDDs, PCDFs) and non-ortho polychlorinated biphenyls (PCBs) for 47 pooled serum samples.

xxx

Section 2: Additional tables and figures

Table S1. Characteristics study population of the Menen ICS and the reference group of FLEHS II (2010-2011).

Characteristic	Flemish Reference Group (*n* = 210)	ICS Menen (*n* = 199)	*p*-Value
% boys	57.6%	57.3%	0.95
Age			<0.01
≤ 14.5y	31.9%	25.1%	
14.5-15.5y	58.6%	45.2%	

> 15.5y	9.5%	29.6%	
BodyMass Index (kg/m²)			<0.01
underweight (<25)	28.1%	32.7%	
normal weight (25-30)	65.7%	62.3%	
overweight (≥30)	6.2%	5.0%	
Smoking			0.67
non-smoker/ex-smoker	91.3%	92.5%	
current smoker	8.7%	7.5%	
Breastfed as a baby			0.23
yes	66.5%	53.8%	
Education participant			0.09
general	48.8%	53.8%	
technical	41.5%	32.0%	
vocational	9.7%	14.2%	
Consumption of locally produced vegetables			<0.01
yes	39.6%	22.2%	
Consumption of locally produced fruit			<0.01
yes	23.3%	4.1%	
Consumption of locally produced chicken eggs			<0.01
yes	45.0%	19.3%	
Consumption of locally caught fish			0.57
yes	8.5%	7.0%	

Table S2. Multiple linear regression results (% change compared to reference category and 95% CI) for PCDD/F-CALUX, dioxin-like PCBs-CALUX, sum 3 PCBs (138 + 153 + 180) and p,p′-DDE in serum (ng/L) for 14-15 year-old adolescents of the FLEHS II study population in the ICS Menen and the Flemish reference group combined (n = 409).

**Covariates**	**PCDD/F-CALUX % Change (95% CI)**	**p-value**	**DL-PCB-CALUX % Change (95% CI)**	***p*-Value**	**Sum 3 PCBs % Change (95% CI)**	***P*-Value**	**p,p′-DDE % Change (95% CI)**	***P*-Value**
**Covariates forced in the models**								
Blood lipids (mg/dL)		**<0.001**		**<0.001**		0.053		0.069
<400	Reference		Reference		Reference		Reference	
400-430	-2.8 (-14.4; +10.5)	0.667	+0.3 (-10.8; +12.7)	0.959	-9.5 (-22.0; +5.1)	0.190	-1.9 (-19.9; +20.1)	0.853
430-480	**-18.2 (-27.8; -7.4)**	**0.002**	**-19.0 (-27.7; -9.3)**	**<0.001**	-4.1 (-17.0; +10.8)	0.565	-17.6 (-32.2; +0.2)	0.053
>=480	**-22.7 (-31.9; -12.2)**	**<0.001**	**-27.5 (-35.4; -18.6)**	**<0.001**	**-18.0 (-29.4; -4.8)**	**0.009**	**-19.3 (-34.0; -1.4)**	**0.036**
Sex		**<0.001**		**<0.001**		**<0.001**		0.100
male	Reference		Reference		Reference		Reference	
female	**-14.7 (-22.0; -6.8)**	**<0.001**	**-16.1 (-22.6; -9.0)**	**<0.001**	**-32.3 (-39.4; -24.4)**	**<0.001**	-11.3 (-23.1; +2.3)	0.100
Age (years)		0.146		**<0.001**		0.334		0.100
<= 14.5	Reference		Reference		Reference		Reference	
14.5-15.5	-7.8 (-16.9; +2.2)	0.121	+5.2 (-4.3; +15.6)	0.292	+5.5 (-7.3; +20.1)	0.416	-10.0 (-23.7; +6.2)	0.213
> 15.5	+1.9 (-11.0; +16.7)	0.781	**+33.1 (+17.6; +50.6)**	**<0.001**	+13.3 (-4.0; +33.8)	0.139	+10.5 (-10.8; +36.9)	0.360
BMI (kg/m²)		0.305		0.274		**<0.001**		**0.007**
underweight (<25)	Reference		Reference		Reference		Reference	
normal weight (25-30)	-3.4 (-12.3; +6.4)	0.483	-5.9 (-13.8; +2.8)	0.180	**-20.5 (-29.0; -10.9)**	**<0.001**	**-18.6 (-30.4; -4.8)**	**0.010**
overweight (≥30)	-15.0 (-31.1; +4.8)	0.107	-11.6 (-27.0; +7.1)	0.208	**-52.2 (-62.4; -39.2)**	**<0.001**	**-35.6 (-53.8; -10.4)**	**0.009**
Smoking		0.158		0.443		0.438		0.481
non-smoker	Reference		Reference		Reference		Reference	
smoker	-10.8 (-24.0; +4.6)	0.158	-5.5 (-18.3; +9.3)	0.443	+8.0 (-11.1; +31.1)	0.438	-9.1 (-30.2; +18.5)	0.481
**Covariates retained at p < 0.05**								

School type participant						**0.001**		
general					Reference			
technical					**-18.7 (-27.6; -8.7)**	**<0.001**		
vocational					**-20.7 (-33.4; -5.7)**	**0.009**		
Breastfed as baby						**<0.001**		**<0.001**
no					Reference		Reference	
yes					**+39.3 (+25.0; +55.3)**	**<0.001**	**+30.7 (+13.1; +51.1)**	**<0.001**
Season						**0.002**		
winter					Reference			
spring					**+25.0 (+10.9; +40.8)**	**<0.001**		
summer					+21.8 (-14.8; +74.3)	0.278		
autumn					+1.7 (-15.0; +21.6)	0.858		
Area of residence		**<0.001**		**0.001**		**<0.001**		**0.006**
Flanders	Reference		Reference		Reference		Reference	
ICS Menen	**-38.3 (-43.8; -32.2)**	**<0.001**	**-14.1 (-21.1; -6.4)**	**0.001**	**-21.6 (-30.3; -11.7)**	**<0.001**	**-19.2 (-30.4; -6.1)**	**0.006**
**Variables concerning locally produced food**								
Model with locally-produced eggs								
Consumption of locally produced eggs		0.267		0.464		**0.003**		**<0.001**
no	Reference		Reference		Reference		Reference	
yes	-5.4 (-14.3; +4.4)	0.267	+3.4 (-5.5; +13.2)	0.464	**+19.2 (+6.2; +33.8)**	**0.003**	**+71.5 (+46.7; +100.4)**	**<0.001**
Model with locally-produced vegetables								
Consumption of locally produced vegetables		0.783		0.778		**0.046**		**<0.001**
no	Reference		Reference		Reference		Reference	
yes	+1.4 (-8.1; +11.7)	0.783	-1.3 (-9.6; +7.8)	0.778	**+12.1 (+0.2; +25.4)**	**0.046**	**+40.1 (+19.9; +63.8)**	**<0.001**
Model with locally-produced fruit								
Consumption of locally produced fruit		0.339		0.082		0.160		0.118
no	Reference		Reference		Reference		Reference	
yes	+6.8 (-6.8; +22.4)	0.339	+11.4 (-1.4; +26.0)	0.082	+11.8 (-4.3; +30.6)	0.160	+19.0 (-4.3; +47.9)	0.118
Model with locally-caught fish								
Consumption of locally caught fish		0.825		0.585		**0.018**		**<0.001**
no	Reference		Reference		Reference		Reference	
yes	-1.9 (-17.0; +16.0)	0.825	-4.1 (-17.7; +11.6)	0.585	**+26.8 (+4.2; +54.3)**	**0.018**	**+86.1 (+41.1; +145.5)**	**<0.001**

PCDD/F-CALUX: polychlorinated dibenzo-p-dioxins and furans activity assessed with the CALUX-essay (Chemically Activated Luciferase gene expression); DL-PCB-CALUX: dioxine-like polychlorinated biphenyls activity assessed with the CALUX essay; sum 3 PCBs: sum of PCB 138, PCB 153 and PCB 180; DDE: dichlorodiphenyldichloroethylene; BMI: body mass index; ICS: industrial contaminated site.

Table S3. Results prioritization of human biomonitoring (HBM) results in the Menen region.

Results PHASE I: Prioritizing HBM Results in Area Menen for Policy Uptake		
**Biomarker**	**Difference Compared to Flemish Reference Values**	**Priority Score (min = 0; max = 60)**
DNA-damage	+26% (comet assay), +69% (oxidative comet assay), +21% 8-oxo-deoxyguanosine	42
PAHs	+49%	55
Thallium in blood	+27%	48
Cadmium in urine	+28%	46
DDE	-30%	36
Dioxins and DL-PCBs	-17% and -39%	29-28
Marker PCBs	-28%	28

DNA: deoxyribonucleic acid; PAHs: polycyclic aromatic hydrocarbons; DDE: dichlorodiphenyldichloroethylene; DL-PCBs: dioxin-like polychlorinated biphenyls.

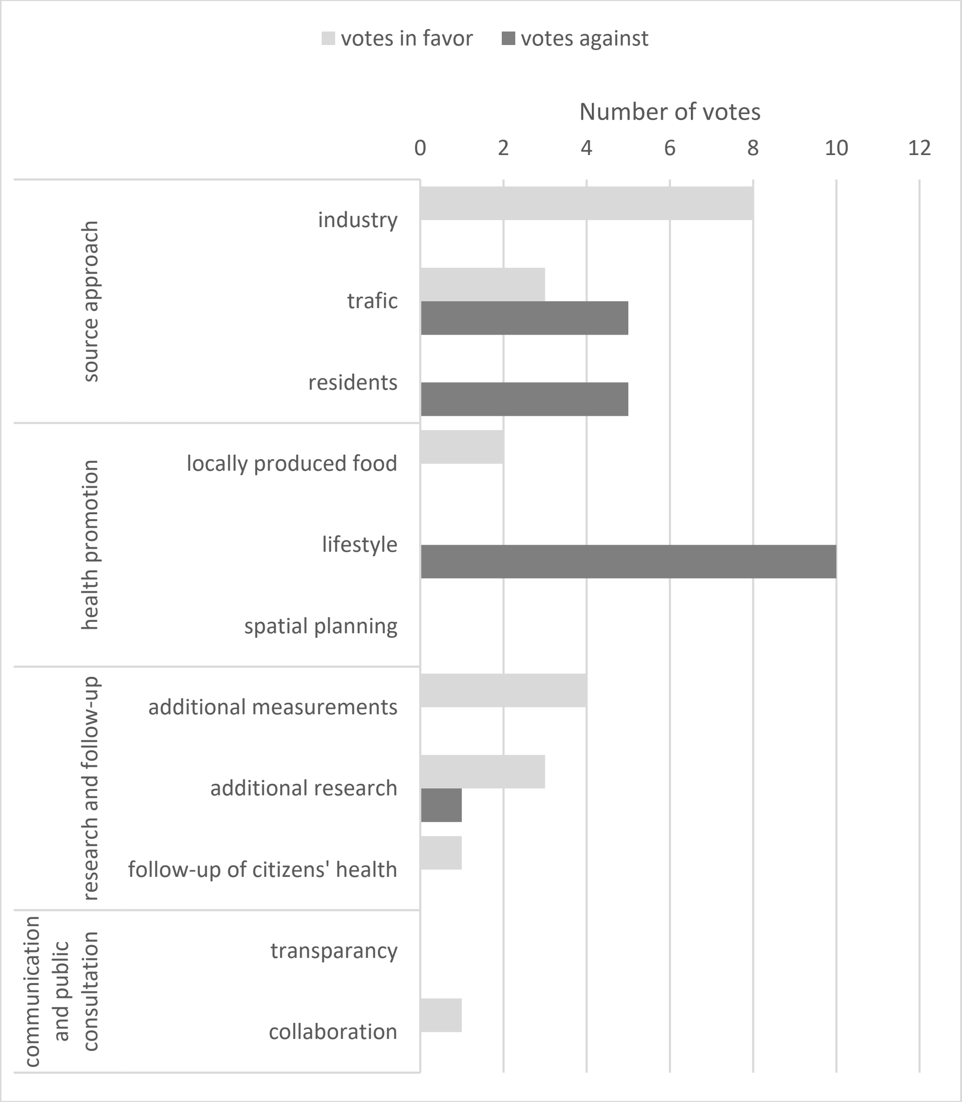

Figure S2. Appreciation of the experts' suggestions by the focus group with participants and their parents.

Table S4. Descriptive characteristics concerning the coops at the 15 locations in the study area in the region of Menen.

Characteristics	Number of locations (%)
Number of chickens	
< 4	6 (40%)
4–6	7 (47%)
> 6	2 (13%)
Size of the coop (m^2)	
≤ 5	6 (40%)
5–15	5 (33%)
> 15	3 (20%)
Degree of vegetation cover in the foraging area	
< ¼	11 (73%)
¼- ½	2 (13%)
½ - ¾	0
> ¾	2 (13%)
Use of insecticides in the coop	4 (27%)
Additional feed for the chickens	
Grass	13 (87%)
Grass clippings	9 (60%)
Weeds	12 (80%)
Table leftovers	15 (100%)
Bread	14 (93%)
Ashes from the stove	0

Table S5: Descriptive statistics for PCDD/Fs, DL-PCBs, marker PCBs and DDT compounds in home-produced eggs from the Menen region.

Pollutant	n> LOQ	LOQ	median	P10	P90
**PCDD/Fs (pg/g lipid weight)**					
2,3,7,8-T4CDD	14	0.091-0.098	0.55	0.39	0.86
1,2,3,7,8-P5CDD	14	0.25-0.27	2.15	1.40	2.40
1,2,3,4,7,8-H6CDD	14	0.17-0.18	1.40	0.77	2.10
1,2,3,6,7,8-H6CDD	14	0.18-0.19	3.05	1.80	4.90
1,2,3,7,8,9-H6CDD	14	0.17-0.18	1.04	0.72	1.40
1,2,3,4,6,7,8-H7CDD	14	0.84-0.91	5.80	2.90	13.00
O8CDD	13	3.33-3.67	15.50	4.00	76.00
2,3,7,8-T4CDF	14	0.12-0.13	3.60	2.80	5.40
1,2,3,7,8-P5CDF	14	0.25-0.27	2.55	2.00	4.70
2,3,4,7,8-P5CDF	14	0.22-0.24	3.55	2.80	5.00
1,2,3,4,7,8-H6CDF	14	0.34-0.36	2.50	1.50	3.80
1,2,3,6,7,8-H6CDF	14	0.34-0.36	2.20	1.20	4.20
1,2,3,7,8,9-H6CDF	0	0.34-0.36	1.95	1.20	3.70
2,3,4,6,7,8-H6CDF	14	0.34-0.36	0.17	0.17	0.18
1,2,3,4,6,7,8-H7CDF	14	0.84-0.91	2.45	1.60	4.70
1,2,3,4,7,8,9-H7CDF	0	0.84-0.91	0.44	0.43	0.46
O8CDF	1	3.33-3.67	1.75	1.70	1.80
**Total PCDD/F**			**53.91**	**29.93**	**117.78**
**DL-PCBs (pg/g lipid weight)**					
PCB 77	14	6.7-7.3	99.0	39.0	270.0
PCB 81	12	3.3-3.7	8.4	1.8	23.0
PCB 126	14	1.701.8	43.5	20.0	110.0
PCB 169	14	1.7-1.8	6.0	2.8	9.7
**Total non-ortho PCB**			**159.4**	**64.1**	**381.9**
PCB 105	14	33.3-36.7	1600.0	720.0	7800.0
PCB 114	14	6.7-7.3	70.5	35.0	390.0

PCB 118	14	66.7-73.3	3500.0	2000.0	17000.0
PCB 123	14	6.7-7.3	93.5	42.0	360.0
PCB 156	14	33.3-36.7	725.0	410.0	2700.0
PCB 157	14	6.7-7.3	150.0	76.0	540.0
PCB 167	14	33.3-36.7	380.0	200.0	1200.0
PCB 189	14	6.7-7.3	58.5	31.0	330.0
**Total mono-ortho PCB**			**6563.0**	**3541.0**	**29690.0**
**Total PCDD/F + DL-PCB**			**6828.07**	**3660.30**	**30256.36**
**Marker PCBs (pg/g lipid weight)**					
PCB 28	13	540-580	870	620	2400
PCB 52	0	540-580	283	275	290
PCB 101	12	540-580	680	285	1200
PCB 138	14	540-580	6700	3800	20000
PCB 153	14	540-580	8550	4700	24000
PCB 180	14	540-580	3950	2200	13000
**Sum marker-PCBs**			**21295**	**12315**	**57380**
**DDT compounds (ng/g lipid weight)**					
o,p′-DDD	0	2	1	1	1
p,p′-DDD	10	2	6	1	24
**Sum DDD**			**7**	**2**	**25**
o,p′-DDE	0	2	1	1	1
p,p′-DDE	14	2	165	52	760
**Sum DDE**			**166**	**63**	**761**
o,p′-DDT	3	2	1	1	10
p,p′-DDT	14	2	49	15	470
**Sum DDT**			**50**	**16**	**483**
**DDD+DDE+DDT**			**229**	**71**	**1301**

PCDD/Fs: polychlorinated dibenzo-p-dioxins and furans activity; DL-PCBs: dioxin-like polychlorinated biphenyls; DDT: dichlorodiphenyltrichloroethane; DDE: dichlorodiphenyldichloroethylene; DDD: dichlorodiphenyldichloroethane.

Table S6. Descriptive statistics for PCDD/Fs, DL-PCBs in home-produced eggs from the Menen region, expressed in pg TEQWHO1998/g lipid weight.

Pollutant (pg TEQWHO1998/g lipid weight)	N> LOQ	median	P10	P90
2,3,7,8-T4CDD	14	0.56	0.39	0.86
1,2,3,7,8-P5CDD	14	2.15	1.40	4.00
1,2,3,4,7,8-H6CDD	14	0.14	0.08	0.21
1,2,3,6,7,8-H6CDD	14	0.31	0.18	0.49
1,2,3,7,8,9-H6CDD	14	0.11	0.07	0.14
1,2,3,4,6,7,8-H7CDD	14	0.06	0.03	0.13
O8CDD	13	0.00	0.00	0.01
2,3,7,8-T4CDF	14	0.36	0.28	0.54
1,2,3,7,8-P5CDF	14	0.13	0.10	0.24
2,3,4,7,8-P5CDF	14	1.78	1.40	2.50
1,2,3,4,7,8-H6CDF	14	0.25	0.15	0.38
1,2,3,6,7,8-H6CDF	14	0.22	0.12	0.42
1,2,3,7,8,9-H6CDF	0	0.20	0.12	0.37
2,3,4,6,7,8-H6CDF	14	0.02	0.02	0.02
1,2,3,4,6,7,8-H7CDF	14	0.03	0.02	0.05
1,2,3,4,7,8,9-H7CDF	0	0.00	0.00	0.01
O8CDF	1	0.00	0.00	0.00
**Total PCDD/F**		**6.27**	**4.36**	**10.32**
PCB 77	14	0.01	0.00	0.03
PCB 81	12	0.00	0.00	0.00
PCB 126	14	4.35	2.00	11.00

PCB 169	14	0.06	0.028	0.10
**Total non-ortho PCB**		**4.42**	**2.04**	**11.11**
PCB 105	14	0.16	0.07	0.78
PCB 114	14	0.04	0.02	0.20
PCB 118	14	0.35	0.20	1.70
PCB 123	14	0.01	0.00	0.04
PCB 156	14	0.36	0.21	1.350
PCB 157	14	0.08	0.04	0.27
PCB 167	14	0.00	0.00	0.01
PCB 189	14	0.01	0.00	0.03
**Total mono-ortho PCB**		**1.00**	**0.54**	**4.29**
**Total PCDD/F + DL-PCB**		**11.641**	**7.150**	**25.473**

PCDD/Fs: polychlorinated dibenzo-p-dioxins and furans activity; DL-PCBs: dioxin-like polychlorinated biphenyls.

Table S7. Descriptive statistics for PCDD/Fs, DL-PCBs in home-produced eggs from the Menen region, expressed in pg TEQWHO2005/g lipid weight.

Pollutant (pg TEQWHO2005/g lipid weight)	n> LOQ	median	P10	P90
2,3,7,8-T4CDD	14	0.56	0.39	0.86
1,2,3,7,8-P5CDD	14	2.15	1.40	4.00
1,2,3,4,7,8-H6CDD	14	0.14	0.08	0.21
1,2,3,6,7,8-H6CDD	14	0.31	0.18	0.49
1,2,3,7,8,9-H6CDD	14	0.11	0.07	0.14
1,2,3,4,6,7,8-H7CDD	14	0.06	0.03	0.13
O8CDD	13	0.01	0.00	0.02
2,3,7,8-T4CDF	14	0.36	0.28	0.54
1,2,3,7,8-P5CDF	14	0.08	0.06	0.14
2,3,4,7,8-P5CDF	14	1.07	0.84	1.50
1,2,3,4,7,8-H6CDF	14	0.25	0.15	0.38
1,2,3,6,7,8-H6CDF	14	0.22	0.12	0.42
1,2,3,7,8,9-H6CDF	0	0.20	0.12	0.37
2,3,4,6,7,8-H6CDF	14	0.02	0.02	0.02
1,2,3,4,6,7,8-H7CDF	14	0.03	0.02	0.05
1,2,3,4,7,8,9-H7CDF	0	0.00	0.00	0.01
O8CDF	1	0.00	0.00	0.00
**Total PCDD/F**		**5.57**	**3.84**	**9.19**
PCB 77	14	0.01	0.00	0.03
PCB 81	12	0.00	0.00	0.01
PCB 126	14	4.35	2.00	11.00
PCB 169	14	0.18	0.084	0.29
**Total non-ortho PCB**		**4.54**	**2.10**	**11.30**
PCB 105	14	0.05	0.02	0.23
PCB 114	14	0.00	0.00	0.01
PCB 118	14	0.11	0.06	0.51
PCB 123	14	0.00	0.00	0.01
PCB 156	14	0.02	0.01	0.08
PCB 157	14	0.01	0.00	0.02
PCB 167	14	0.01	0.01	0.04
PCB 189	14	0.00	0.00	0.01
**Total mono-ortho PCB**		**0.20**	**0.11**	**0.89**
**Total PCDD/F + DL-PCB**		**10.21**	**6.09**	**21.55**

PCDD/Fs: polychlorinated dibenzo-p-dioxins.

Section 3: The local action plan for the region of Menen

As presented in the main article, a local action plan was co-created after the second human biomonitoring study (FLEHS II (2007-2011) in the region of Menen, involving scientists of the study, the regional authorities, and representatives of the local community (citizens, local authorities, and other local stakeholders). Perspectives and opinions of citizens and local authorities were taken into account in this action plan. The citizens mainly asked for source control and better communication, but also acknowledged the relevance of (temporary) behavior change to reduce exposure. The action plan consisted of 16 different actions.

1) Conducting research on PCDD/Fs, PCBs and DDE in samples of local soil and home-produced food and active communication of the results
2) Providing information on healthy gardening for citizens
3) Making maximum use of the advisory role of the governmental bodies engaged in the action plan, with regard to traffic impact when re-permitting existing companies or environmental permits of new companies
4) Decreasing local traffic impact by setting conditions for the further expansion of industrial areas and by conducting research on local sources of polycyclic aromatic hydrocarbons (PAHs)
5) Conducting research on the relative importance of different exposure routes to PAHs in the region of Menen to identify additional mitigating actions
6) Estimation of the health-based risk of thallium exposure to formulate policy recommendations for further monitoring in the environment and in humans
7) Exploring the need for further follow up and specify specific follow up scenarios for the region of Menen
8) Appointing general practitioners as a local contact center for medical-environmental questions
9) Establishment of a digital and physical information center, as part of integrated and targeted communication actions
10) Establishing a joint communication structure between all local actors involved
11) Signing and executing the communication charter that resulted from the AEROPA-project
12) Development of an online dictionary in which complex, technical or official concepts are clearly and concisely explained
13) Companies engage in awareness raising of their employees on the environment, health and safety in the interest of the employees and in the interest of the local residents
14) Introducing the educational package on heavy metals in kindergartens and primary schools in the region of Menen
15) The city of Menen is conducting an awareness raising campaign on indoor and outdoor burning
16) Further fine-tuning the local action plan in time, in co-production with local stakeholders and citizens, with opportunities for the development of new actions and/or initiatives in order to achieve a healthier environment in the region of Menen
